# Photon beam modeling variations predict errors in IMRT dosimetry audits

**DOI:** 10.1016/j.radonc.2021.10.021

**Published:** 2021-11-05

**Authors:** Mallory C. Glenn, Fre’Etta Brooks, Christine B. Peterson, Rebecca M. Howell, David S. Followill, Julianne M. Pollard-Larkin, Stephen F. Kry

**Affiliations:** a Department of Radiation Oncology, University of Washington, Seattle;; b Department of Radiation Physics, The University of Texas MD Anderson Cancer Center;; c The University of Texas MD Anderson Cancer Center UTHealth Graduate School of Biomedical Sciences;; d Department of Biostatistics, The University of Texas MD Anderson Cancer Center, Houston, United States

**Keywords:** Phantoms, Dosimetry audit, Treatment planning system, Beam modeling

## Abstract

**Background & purpose:**

To evaluate treatment planning system (TPS) beam modeling parameters as contributing factors to IMRT audit performance.

**Materials & methods:**

We retrospectively analyzed IROC Houston phantom audit performance and concurrent beam modeling survey responses from 337 irradiations performed between August 2017 and November 2019. Irradiation results were grouped based on the reporting of typical or atypical beam modeling parameter survey responses (<10th or >90th percentile values), and compared for passing versus failing (>7% error) or “poor” (>5% error) irradiation status. Additionally, we assessed the impact on the planned dose distribution from variations in modeling parameter value. Finally, we estimated the overall impact of beam modeling parameter variance on dose calculations, based on reported community variations.

**Results:**

Use of atypical modeling parameters were more frequently seen with failing phantom audit results (*p* = 0.01). Most pronounced was for Eclipse AAA users, where phantom irradiations with atypical values of dosimetric leaf gap (DLG) showed a greater incidence of both poor-performing (*p* = 0.048) and failing phantom audits (*p* = 0.014); and in general, DLG value was correlated with dose calculation accuracy (*r* = 0.397, p < 0.001). Manipulating TPS parameters induced systematic changes in planned dose distributions which were consistent with prior observations of how failures manifest. Dose change estimations based on these dose calculations agreed well with true dosimetric errors identified.

**Conclusion:**

Atypical TPS beam modeling parameters are associated with failing phantom audits. This is identified as an important factor contributing to the observed failing phantom results, and highlights the need for accurate beam modeling.

In radiation therapy, it is necessary that patients receive the intended dose in the intended location. Shortcomings in radiation therapy, i.e. deviations from a protocol-defined radiation dose, have been associated with a dramatic decrease in overall survival. For example, deviations from protocol decreased rates of 2-year overall survival or local tumor control by 20% among head and neck patients [1]. Similar meta-analyses found that protocol deviations were associated with a 75% increase in the risk of treatment failure and mortality [2].

Dosimetry audits have long been an invaluable tool to validate the dosimetric accuracy for both everyday radiotherapy practice and clinical trials. One such tool is the anthropomorphic phantom audit through the Imaging and Radiation Oncology Core Houston Quality Assurance Center (IROC Houston). IROC Houston offers end-to-end quality assurance to radiotherapy facilities around the world through its anthropomorphic phantoms to evaluate that what is described by the treatment planning system (TPS) agrees with the delivered dose. In this program, institutions irradiate an IROC Houston phantom containing thermoluminescent dosimeters (TLDs) and radiochromic film, whose dose measurements are then compared against the institution’s calculated dose [3–6]. Irradiations either pass or fail to meet acceptance requirements depending on whether measured and calculated TLD doses agree within 5–7% and films yield ≥85% of pixels passing 5%/3mm to 7%/4mm gamma criteria, depending on the phantom test.

Currently, 8–15% of phantom irradiations fail to meet these relatively loose acceptance criteria [7–9]. Patterns have been identified among failing phantoms. For the head and neck (H&N) phantom, IROC’s most widely irradiated phantom, failures are mostly caused by systematic errors in dose; that is, the dose is administered in the correct location, but with systematically the wrong magnitude [7].

The cause of these dosimetric errors is of critical interest and concern given their frequency and potential effects on patient care and outcomes. Recent evaluations have identified that many poorly-performed irradiations are associated directly with errors in the TPS dose calculation [10,11]. However, substantial questions remain about which aspect(s) of dose calculations are suspect.

Recently, IROC Houston identified substantial variations in how radiotherapy institutions model their clinical beams for linear accelerators (linacs) of the same model, beam energy, and type of multi-leaf collimator (MLC), particularly with respect to parameters describing MLC characteristics [12]. These disparities are even more suspect given that many of today’s linacs perform similarly dosimetrically [13,14]. Previous studies have underscored substantial dosimetric impact from variations in certain TPS parameters, including the dosimetric leaf gap in Eclipse (Varian Medical Systems, Palo Alto, CA) or MLC offset in RayStation (RaySearch Laboratories, Stockholm, Sweden) [15–19]. Thus, examining phantom performance in the context of an institution’s beam modeling parameter choices is of interest.

The goal of this study was to investigate potential relationships between an institution’s reported beam modeling and the accuracy of their phantom dosimetry audit. Understanding the link between treatment plan performance and specific choices of beam model parameter values can help expose the cause of the frequent errors seen in IROC phantoms and other dosimetry audits. Characterizing TPS-related errors on a multi-institutional scale will lead to improved resolution of suboptimal IROC phantom results, increases in dosimetric accuracy, and associated improvements in patient outcomes, especially in the context of clinical trials.

## Methods

We reviewed 337 IMRT phantom audits from IROC Houston between August 2017 to November 2019, along with concurrently reported TPS beam modeling parameters associated with each irradiation. All audits were performed independently by the institution using their clinical beam model and planning and treatment processes. Because of the contemporary nature of this data, the results of this work are up-to-date and reflect current treatment equipment, TPS model commissioning, and treatment strategies. Phantoms examined in this work were the H&N, prostate, and spine phantoms. Each phantom contains unique challenges for planning and delivery, including multiple target volumes (e.g. H&N phantom) and avoidance structures for which IMRT or VMAT are necessary to treat appropriately. These phantoms do not move and have previously been identified by IROC Houston as having major error modes related to systematically under- or overdosing the targets [7,8].

### Relationships between TPS beam modeling parameters and phantom audit result

We first examined if an institution’s use of atypical beam modeling parameter values was associated with poor or failing audit results. Atypical parameter values were specific to the TPS, linac class (e.g., Varian TrueBeam), beam energy, and MLC type, and were assessed by comparison to published community values from a TPS beam modeling parameter survey administered by IROC Houston [12]. Table 1 describes one set of the parameters collected in this survey, as well as the range of dose effects each parameter exhibits in the IROC head and neck phantom, based on the variations reported by Glenn et al. [20] Phantom irradiations were grouped based on the presence of atypical parameter values. Here, “atypical” was defined as the outer 20% of the survey responses, being either <10th percentile or >90th percentile compared to the community values (for the same linac type, beam energy, and MLC make and model). All TPS parameters of interest were examined individually. These groups of irradiations with either typical or atypical beam modeling parameters were compared using Fisher’s exact test to determine if the proportions of institutions adopting atypical values were different among passing and failing phantom irradiations (those having dose errors greater than 7%). Likewise, this analysis was performed for each parameter by comparing well-performed and poorly-performed irradiations; poorly performing phantom audits may still be within tolerance, but have at least one TLD measurement that differs by more than 5% from the TPS-calculated dose. Finally, we looked at relationships between individual parameter values and phantom performance accuracy.

These evaluations were conducted on the complete data set of all 337 phantoms, including all machine, TPS, MLC, and beam energy combinations.

### Impact of beam modeling variations on dose distributions

We evaluated the impact of atypical beam modeling parameter values on the dose distribution in IROC phantoms, in particular, the dose profiles through the target volumes. These results were then qualitatively compared to actual errors observed in the community. We designed beam models in Eclipse with the AAA algorithm and in RayStation to represent a standard 6 MV Varian Base class [13] accelerator (e.g. Trilogy, 2100iX, etc.) equipped with Milennium120 MLC with average modeling characteristics [12]. This class was chosen because it had the most collectively robust statistics (i.e. survey samples) across the two TPS environments and serves as an example of what the dose impact is from changes in the TPS parameters. We then evaluated changes in dose on five clinically-acceptable IMRT H&N phantom plans after individually modifying the parameters in Table 1 according to their reported distributions [12] (all plans used fixed MUs). Values for the parameters ranged from the 2.5th to 97.5th percentiles in order to encapsulate the greatest extent for potential changes in dose distributions related to beam modeling choices. Changes in dose distributions were compared with IROC Houston phantom irradiation results to assess qualitative similarities.

### Estimating TPS dose calculation errors based on community data

Glenn et al. [20] previously showed that as TPS parameter values in Eclipse and RayStation were varied (those values shown in Table 1), the dose distribution changed linearly with parameter value, and this dose variation was independent of plan type (VMAT or IMRT). Moreover, parameter effects were independent and not influenced by the values used for other parameters within the TPS [20]. A range of estimated dose contributions, based upon community reported data, is included in Table 1, using median parameter values as the baseline. Based on these characteristics, we predicted the expected dosimetric deviations caused by beam modeling variations for the IROC Houston phantom irradiations performed with a Varian Base class accelerator. For example, if the DLG value used by one institution was at the 90th percentile, we expect this to cause an overestimation of predicted dose of 1.2%. Similarly, if their MLC transmission was at the 80th percentile, we expect this to cause an overestimation of 0.2%. Because these factors are independent, we can estimate an aggregate TPS error of 1.4% in this phantom audit. For each phantom result, this aggregate predicted TPS error was determined for all parameters, and then compared with the actual phantom error that was measured for that institution. These results were evaluated using the Pearson correlation between estimated and true dose errors observed. In order to isolate cases for which dose errors were the main contributor to phantom performance, and thus best test whether dose error contributions could be estimated, we excluded 11 phantom cases that exhibited localization errors of greater than 3 mm (identified by measured film profiles).

Unlike the methods in Section “Relationships between TPS beam modeling parameters and phantom audit result”, this analysis requires evaluation of the dosimetric impact of TPS parameter variations. Because an abundance of data was for the Varian Base class with a Millennium 120 MLC and delivered with a 6 MV beam, and because we had previously evaluated the dosimetric impact of TPS parameter variations on this combination [20], we focused exclusively on this combination, and this subset of irradiations, for predicting errors.

## Results

Table 2 summarizes the breakdown of the 337 phantom irradiations examined. The majority of irradiations were performed on Varian machines (86%) using Eclipse as the primary TPS (78%). Most irradiations (77%) were performed on the IROC H&N phantom. 31 phantoms (9.2%) failed to meet credentialing criteria (±7% TLD dose and 7%/4 mm gamma criteria for film) and 57 (16.9%) exhibited poor performance (at least one measurement outside ±5%).

### Relationship between TPS parameters and phantom performance

For the 337 irradiations, atypical parameter values were identified in 68% of failing cases (*n* = 19) and 56% of poorly performed irradiations (*n* = 30). In contrast, atypical parameter values were present in only 42% of well-performed irradiations (*n* = 113). These proportions were statistically different, with atypical parameters consistently present more often in failing irradiations (Chi-Square, *p* = 0.01). Additionally, when examining only parameters that caused potential changes in dose greater than 1% (Table 1), 57% of failing irradiations and 50% of poorly performed cases reported atypical parameters, while only 32% of well-performed irradiations reported atypical values in these impactful parameters. Impactful atypical values were significantly more associated with failing irradiations (Chi-Square, *p* = 0.007).

When evaluating specific TPS parameter values, atypical values of dosimetric leaf gap (DLG) in Eclipse AAA were related to poor performance (*p* = 0.048, Fisher’s Exact test) and failing irradiations (*p* = 0.014), consistently occurring more often with each negative outcome than those using more typical DLG values. A low but significant Pearson’s correlation coefficient was identified between Eclipse DLG percentile score and average TLD dose calculation error for all applicable phantoms examined (*r* = 0.293, p < 0.001); that is, using a larger DLG value, as compared to the community consensus, was associated with overestimating the dose to the phantom. Interestingly, for Eclipse AAA beam models, values used for DLG and MLC transmission factor were positively correlated (*r* = 0.615, p < 0.001), meaning users assigning a higher than typical DLG were more likely to use a greater value for the MLC transmission factor as well, compounding potential dose errors.

The only other relationship observed among specific parameters was that the primary source X width in RayStation was related to poorly performed (*p* = 0.007) and failing irradiations (*p* = 0.042). However, this result was based on dramatically fewer cases than observed with Eclipse users, is physically suspect because the source Y width did not show this trend, and is inconsistent with previous findings [20] that showed this parameter did not substantially affect the dose calculation (Table 1); consequently, this relationship requires further investigation.

### Impact of beam modeling parameters on dose distribution

**Fig. 1.**
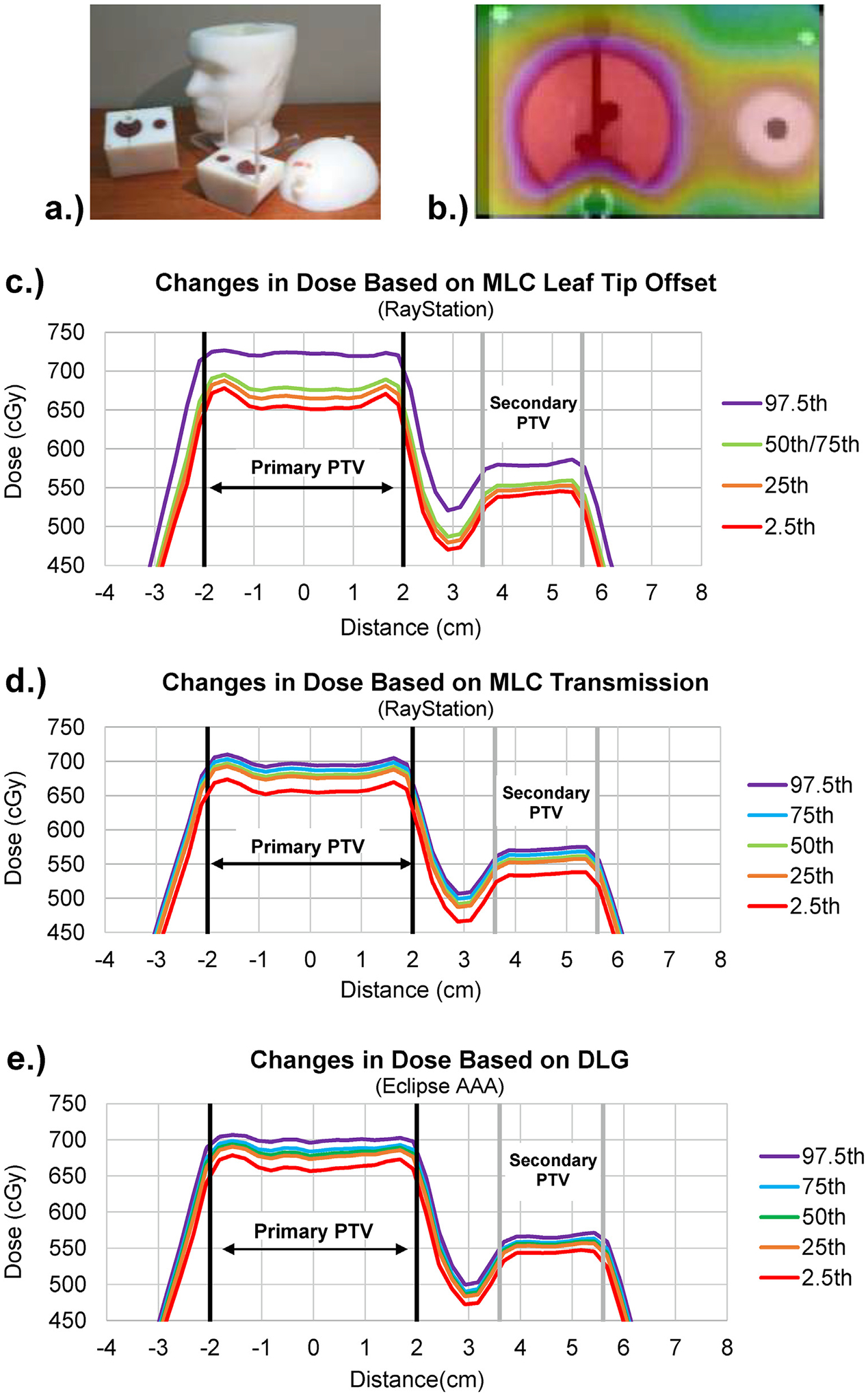
Dose profiles for the (a) IMRT H&N phantom with (b) a typical planar dose distribution in the phantom covering the primary and secondary planning target volumes (PTV). Changes in plan dose were calculated for the primary and secondary PTVs following manipulation of (c) RayStation MLC leaf tip offset, (d) RayStation MLC transmission factor, and (e) Eclipse dosimetric leaf gap (DLG) for an IMRT H&N phantom plan based on 2.5th to 97.5th percentiles of beam modeling survey results.

H&N phantom treatment plans were recalculated with different beam modeling parameter values for the Varian base class beam model and resulted in systematic changes in dose. Fig. 1 shows the phantom, a typical dose distribution, and visualizes such dose changes for some parameters of interest for a 9-field IMRT plan, showing the same shape of dose distribution but systematically offset as the TPS parameter changes. This pattern remained true for all parameters listed in Table 1. Like the ranges of potential dose contributions in Table 1, changing parameters representing the MLC leaf-tip offset (i.e. dosimetric leaf gap in Eclipse and the MLC position offset in RayStation) produced the greatest magnitude of dose changes, often surpassing 5% for atypical values used in the community. Parameters representing the source size, MLC gain, and MLC curvature produced no changes in the resultant dose distributions, consistent with previous observations [20].

**Fig. 2.**
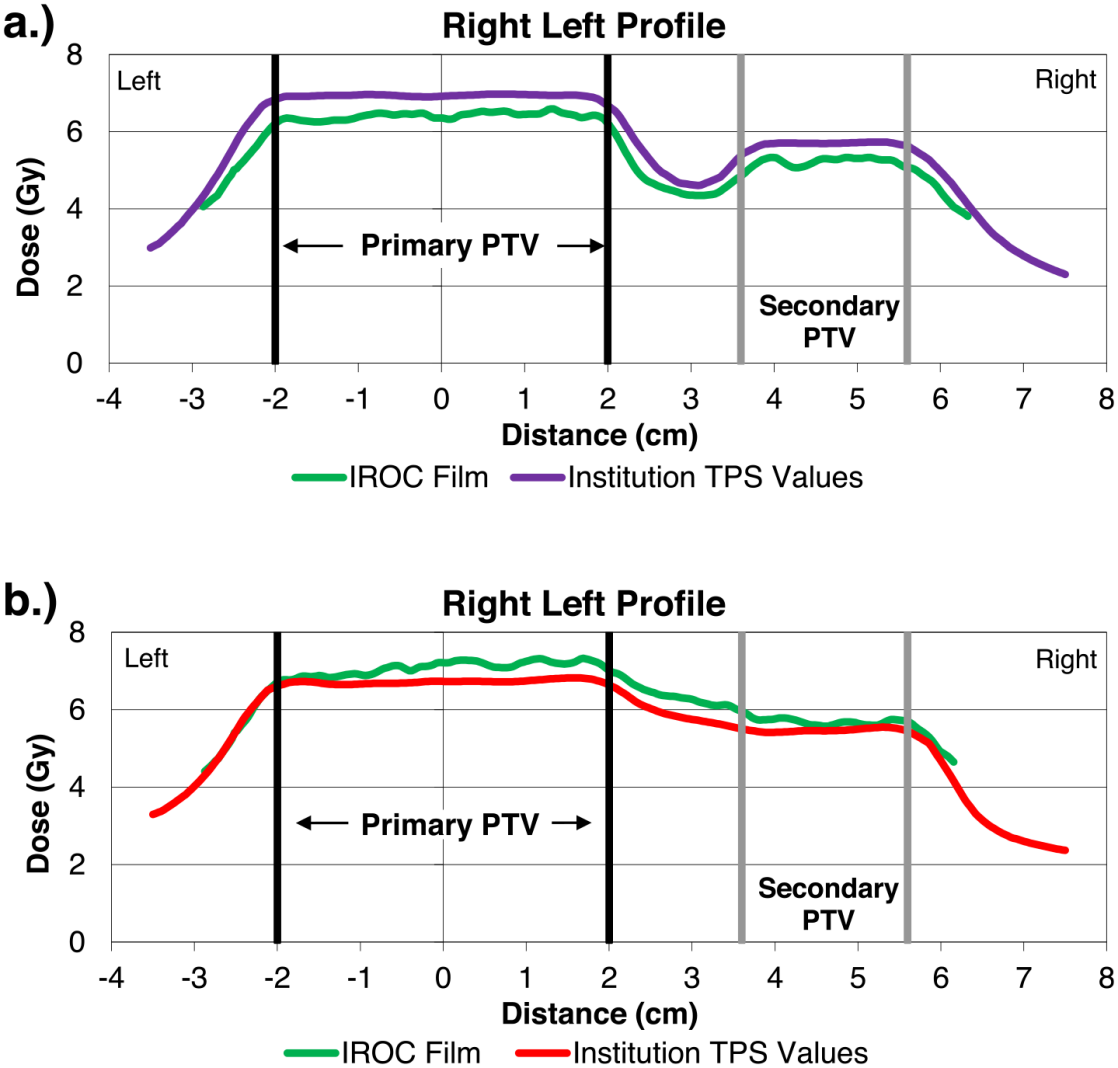
Phantom cases of interest. (a) Irradiation using Eclipse AAA with high dosimetric leaf gap (91st percentile) that overestimated the dose delivered (purple) compared to film measurement (green) by ~ 7%. The institution overestimated the dose on a second attempt three months later using the same beam model. (b) Irradiation using Eclipse AAA with very low DLG (1st percentile) and MLC transmission (2.5th percentile), that underestimated the dose delivered (red) compared to film measurement (green) by 5% on average. A second attempt was made after adjusting the DLG (from 0.06 cm to 0.125 cm), which improved the accuracy substantially and resulted in a passing audit.

The differences in dose calculations shown in Fig. 1 qualitatively match the most common form of phantom errors that are observed: systematic dose errors [7]. Moreover, evaluation of previous phantom audits revealed cases that display these type of systematic dose deviations when atypical values were used. Examples of this are shown in Fig. 2, which shows right-left film plane measurements of two previous H&N phantom irradiations compared to TPS-calculated dose profiles for the same regions. In Fig. 2.a, the institution’s beam model used an atypical value for DLG (91st percentile) and systematically overestimated the dose by approximately 7%. To the opposite effect, Fig. 2.b depicts dose profiles for an institution whose beam model used atypically low values for DLG and MLC transmission (1st and 2.5th percentile, respectively) that subsequently underestimated the delivered dose by 5% on average.

### Estimating dose error

**Fig. 3.**
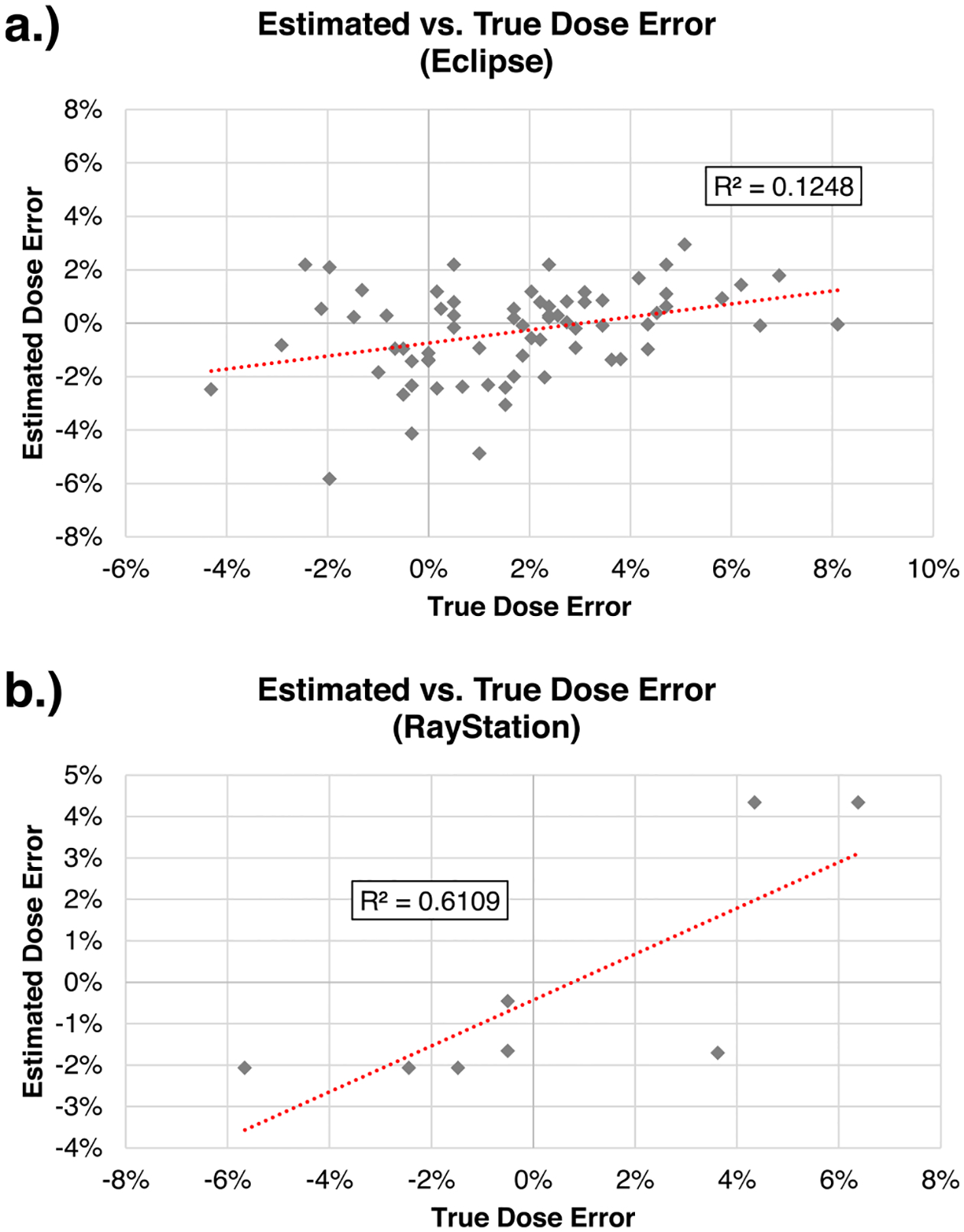
Comparison of estimated versus true dosimetric error for phantom irradiations performed using a Varian Base class Linac12 for (a) Eclipse and (b) RayStation. Phantom irradiations with setup errors greater than 3 mm were excluded (as identified by measured film profiles).

Phantom irradiations performed with a Varian Base class linac and having no identifiable localization issues (verified by film measurement) were selected to test the potential for estimating dosimetric errors caused by beam modeling parameter variations. Total estimated dose errors, defined as the sum of individual dose error contributions from each investigated parameter, were correlated with phantom dose inaccuracy for both Eclipse (*r* = 0.353, *p* = 0.003) and RayStation (*r* = 0.782, *p* = 0.022) as shown in Fig. 3.

## Discussion

Our study highlights that TPS modeling parameters are frequently involved in failing IROC phantom irradiations. While it is known that poor beam modeling will result in poor dose calculation accuracy, this work reveals that this is a critical failure mode that is currently and substantially affecting the radiotherapy community. As mentioned in the introduction, an unacceptably high fraction of institutions fail to accurately deliver dose to IROC’s phantoms (8–15%). The current study reveals that poor beam modeling, as indicated by use of atypical beam modeling parameters, is a substantial driving cause of these errors in dose delivery.

First, there exist clear relationships between beam modeling parameter value and phantom outcome. The DLG, for example, was an important parameter in the dose calculation; while this finding has been known [16,17,19], in this work we show that atypical modeling of this parameter is directly associated with poor performance on IMRT phantom audits. While a significant correlation was found, it is clear from Fig. 3 that the predicted dose error does not capture the entire difference between measurement and TPS calculation. This result is unsurprising; there exist other error modes that are not accounted for in this evaluation, such as Hounsfield unit-to-density curve errors, input dosimetric beam data errors, machine delivery uncertainties, calibration errors, among others. In addition, different linacs may appropriately have different TPS parameters (i.e., the optimal DLG will be different for different machines), whereas our analysis considers only the mean TPS value as the baseline. Despite the simple assumptions in our approach, these estimations nevertheless accounted for a reasonable portion of observable error (for example, estimating +4% error when the true TLD dose error was +6%). This indicates that dose calculation errors are identifiable and contribute substantially to errors occurring in irradiations of IROC phantoms.

Second, when manipulating beam models, the variation of any single parameter produced systematic changes in dose across all regions in the phantom. These results directly parallel the observations of Carson et al [7] and Edward et al. [22] which identified systematic dose discrepancies as the predominant cause of poor IROC audit performance among non-moving phantoms. The examples of actual phantom audit failures in Fig. 2 demonstrate the same pattern. These irradiations exhibit atypical beam modeling and show the potential for poor outcomes in what the institution believed to be clinically-acceptable treatment plans. To date, origins for these poor phantom irradiations have not been well-understood, but the current work highlights several highly-probable causes related to beam modeling.

An unsettling tangential observation also arose from this work: for all 337 plans in this cohort, the institution self-reported passing their institutional pre-treatment IMRT QA. For the cases shown in Fig. 2, which showed systematic dose differences of 7% and 5% respectively, the institutions reported 96% or more pixels passing for every field evaluated using 3%/3 mm gamma analysis, absolute dose mode, and a 20% low dose threshold, as measured by portal dosimetry. For these examples, IMRT QA was insufficient in identifying problematic plans that showed systematic and understood dose errors. These results reinforce other studies that have shown that IMRT QA is insensitive at identifying unacceptable plans [21,23–28]. Consequently, beam modeling inaccuracies, as identified in the current study, are difficult to detect using conventional IMRT QA methods, even though they can have clinical consequences on dose accuracy [15,24].

This analysis demonstrates that using parameter values that are not extreme can lead to more accurate representation of linac performance. However, this work is limited in that it is in the context of a single, common linac class; dose error estimations of other popular linacs could not be assessed due to more limited statistics for other linac/TPS combinations. However, we expect that these trends could be generalizable to different linac classes given the proclivity for atypical modeling to be associated with poor phantom performance, regardless of machine type. Furthermore, these dose error estimations only consider beam modeling as a contributing factor to phantom performance, which cannot fully represent errors observed in static phantoms (e.g., with further characterization of other error modes, these estimations, as well as recommendations for improvement, could be improved).

Though we identified that atypical parameter values were associated with poorer audit performance, the data do not advocate for a universal, standard beam model. For those failing irradiations that reported using atypical parameter values, especially the DLG or MLC offset, it is extremely likely that changing these parameters to more typical values could have resulted in passing the phantom audit. However, there were also cases for which an atypical parameter value coincided with highly accurate delivered dose. Such well-performing models describe non-reference machines, and dosimetric accuracy could suffer dramatically if reference values were used for such cases. In short, while the median value is likely a good fit, it is essential to use values that are appropriate for the system and its intended use. Values implemented into a beam model need to be scrutinized and tested for robustness under a variety of clinical scenarios before determining their suitability. Given the performance of current IMRT QA tools, and consistent with AAPM MPPG5.a. [29], this should include independent evaluation.

## Conclusion

This study examined relationships between beam modeling parameter values used by the radiotherapy community and IROC phantom audit performance. Notable correlations were identified between atypical DLG values and overall phantom performance. In general, atypical TPS parameter values were directly correlated with actual delivery errors in the IROC phantoms, but this could not describe all cases. Specific beam model parameters, especially those that represent the MLC characteristics, were found to be substantially involved in failing dosimetry audits. Of note, variations in these parameters manifested as systematic discrepancies between calculated and measured dose. These dosimetric discrepancies could be estimated and could account for a substantial portion of dosimetric errors in phantom audits.

Given the high frequency of dosimetry audit failures, these results underscore a major problem related to radiotherapy quality and point to specific factors as potential causes. As such, this work provides direct guidance to physicists who receive suboptimal results on how to improve the quality of their radiotherapy. More broadly, these results demand increased attention to these modeling parameters during the commissioning of treatment planning systems. These efforts have the potential to reduce the frequency of failed audits and improve the quality of radiotherapy across the broader community.

## Figures and Tables

**Table 1:** Treatment planning system beam modeling parameters requested via IROC Houston surveys and their range of dose effects (based on the reported spread in values), as previously determined by phantom dose calculations, for a common base Varian linac model equipped with Millennium120 MLC (e.g. Trilogy, 2100iX, etc.) using 6 MV photons [13,20].

TPS Parameter	Estimated Dose Effects							
	*2.5th Percentile*		*10th Percentile*		*90th Percentile*		*97.5th Percentile*	
	Parameter Value	Dose Effect (vs. 50th percentile)	Parameter Value	Dose Effect (vs. 50th percentile)	Parameter Value	Dose Effect (vs. 50th percentile)	Parameter Value	Dose Effect (vs. 50th percentile)
***Eclipse AAA***								
Effective Target Spot Size X and Y [mm]	0.0000	0.0%	0.0000	0.0%	0.5000	0.0%	1.0000	0.0%
MLC Transmission Factor	0.0118	−1.1%	0.0134	−0.7%	0.0200	+0.8%	0.0200	+0.8%
Dosimetric Leaf Gap [cm]	0.1000	−3.6%	0.1388	−1.5%	0.2000	+1.2%	0.2300	+2.8%
***RayStation***								
Primary Source X Width and Y Width [cm]	0.05000	0.0%	0.04000	0.0%	0.09700	0.0%	0.12345	0.0%
MLC Transmission	0.0070	−4.0%	0.0070	−4.0%	0.0250	+2.3%	0.0250	+2.3%
Tongue and Groove [cm]	0.0100	+1.1%	0.0100	+1.1%	0.0500	−0.3%	0.0500	−0.3%
Leaf Tip Width [cm]	0.1770	−1.6%	0.1860	−1.4%	0.5000	+1.9%	0.5000	+1.9%
MLC Position Offset [cm]	0.0000	−3.6%	0.0000	−3.6%	0.1160	+6.7%	0.1160	+6.7%
MLC Position Gain	0.0000	0.0%	0.0000	0.0%	0.0150	0.0%	0.0150	0.0%
MLC Position Curvature [1/cm]	0.0000	0.0%	0.0000	0.0%	0.0010	+0.2%	0.0010	+0.2%

**Table 2:** Summary of phantom irradiations.

Category	*N*	(%)
***TPS***		
Eclipse (AAA)	226	67.1%
Eclipse (AcurosXB)	38	11.3%
Pinnacle	40	11.9%
RayStation	33	9.8%
***Linac types***		
Varian Base Class (Clinac series)	116	34.4%
Varian TrueBeam	165	49.0%
Elekta Agility (VersaHD, etc.)	40	11.9%
Other	16	4.7%
***Beam energy***		
6 MV	287	85.2%
6 FFF	22	6.5%
10 MV	21	6.2%
10 FFF	6	1.8%
15 MV	1	0.3%
***Phantom***		
Head and neck	258	76.6%
Spine	34	10.1%
Prostate	45	13.4%
